# *Trypanosoma cruzi* Evades the Complement System as an Efficient Strategy to Survive in the Mammalian Host: The Specific Roles of Host/Parasite Molecules and *Trypanosoma cruzi* Calreticulin

**DOI:** 10.3389/fmicb.2017.01667

**Published:** 2017-09-01

**Authors:** Galia Ramírez-Toloza, Arturo Ferreira

**Affiliations:** ^1^Laboratory of Parasitology, Department of Animal Preventive Medicine, Faculty of Veterinary Medicine and Livestock Sciences, University of Chile Santiago, Chile; ^2^Program of Immunology, Institute of Biomedical Sciences, Faculty of Medicine, University of Chile Santiago, Chile

**Keywords:** *Trypanosoma cruzi*, complement system, immune evasion of parasites, complement regulatory proteins, *Trypanosoma cruzi* calreticulin

## Abstract

American Trypanosomiasis is an important neglected reemerging tropical parasitism, infecting about 8 million people worldwide. Its agent, *Trypanosoma cruzi*, exhibits multiple mechanisms to evade the host immune response and infect host cells. An important immune evasion strategy of *T. cruzi* infective stages is its capacity to inhibit the complement system activation on the parasite surface, avoiding opsonizing, immune stimulating and lytic effects. Epimastigotes, the non-infective form of the parasite, present in triatomine arthropod vectors, are highly susceptible to complement-mediated lysis while trypomastigotes, the infective form, present in host bloodstream, are resistant. Thus *T. cruzi* susceptibility to complement varies depending on the parasite stage (amastigote, trypomastigotes or epimastigote) and on the *T. cruzi* strain. To avoid complement-mediated lysis, *T. cruzi* trypomastigotes express on the parasite surface a variety of complement regulatory proteins, such as glycoprotein 58/68 (gp58/68), *T. cruzi* complement regulatory protein (TcCRP), trypomastigote decay-accelerating factor (T-DAF), C2 receptor inhibitor trispanning (CRIT) and *T. cruzi* calreticulin (TcCRT). Alternatively, or concomitantly, the parasite captures components with complement regulatory activity from the host bloodstream, such as factor H (FH) and plasma membrane-derived vesicles (PMVs). All these proteins inhibit different steps of the classical (CP), alternative (AP) or lectin pathways (LP). Thus, TcCRP inhibits the CP C3 convertase assembling, gp58/68 inhibits the AP C3 convertase, T-DAF interferes with the CP and AP convertases assembling, TcCRT inhibits the CP and LP, CRIT confers ability to resist the CP and LP, FH is used by trypomastigotes to inhibit the AP convertases and PMVs inhibit the CP and LP C3 convertases. Many of these proteins have similar molecular inhibitory mechanisms. Our laboratory has contributed to elucidate the role of TcCRT in the host-parasite interplay. Thus, we have proposed that TcCRT is a pleiotropic molecule, present not only in the parasite endoplasmic reticulum, but also on the trypomastigote surface, participating in key processes to establish *T. cruzi* infection, such as inhibition of the complement system and serving as an important virulence factor. Additionally, TcCRT interaction with key complement components, participates as an anti-angiogenic and anti-tumor molecule, inhibiting at least in important part, tumor growth in infected animals.

## Introduction

Chagas’ disease or American Trypanosomiasis represents a serious burden for millions of people worldwide. This parasitic infection, considered a neglected reemerging tropical disease, causes more than 10,000 deaths each year, being a main cause of heart failure in Latin America, where it is endemic (Ferreira et al., 2016). However, Chagas disease is currently expanding to non-endemic countries (United States and Canada) and continents (Europe, Asia, and Oceania) due to migration of infected people (Sbaraglini et al., 2016), affecting nearly 6 million people worldwide (WHO, 2015).

*Trypanosoma cruzi*, the etiological agent of Chagas disease, exhibits a variety of mechanisms to evade the host immune response. These mechanisms enable the parasite to establish an infection and persist in the host, determining a chronic stage (Freire-de-Lima et al., 2016). The *T. cruzi* life-cycle is extremely complex, involving an invertebrate hematophagous triatomine vector and an extensive range of mammalian hosts, including humans (Chacón et al., 2016). *T. cruzi* metacyclic trypomastigotes, one of the infective forms of the parasite, are released in the feces or urine of triatomines after a blood meal. These infective forms encounter mucosa or discontinuous regions of the epithelium, infecting mammalian host cells (Cardoso et al., 2015). To invade these cells, trypomastigotes use a variety of virulence factors that interact with host components to help the parasite to invade mammalian cells. In trypomastigotes, gp90 (Dorta et al., 1995; Yoshida, 2006), mucins (Villalta and Kierszenbaum, 1984; Yoshida et al., 1989), Tc85, trans-sialidase family (gp85, gp82 and TSA-1) (Cross and Takle, 1993), gp35/50 (Teixeira and Yoshida, 1986), TS (Previato et al., 1985), gp83 (Lima and Villalta, 1988; Villalta et al., 2008); penetrin (Ortega-Barria and Pereira, 1991); and proteases such as cruzipain, oligopeptidase B and Tc80 (Murta et al., 1990), have been described. Additionally, we have shown that *T. cruzi* calreticulin (TcCRT) is an important virulence factor on the *T. cruzi* surface (Ramirez et al., 2011b).

In the host cell, trypomastigotes are confined in a parasitophorous vacuole, from which they escape to differentiate into amastigotes, the replicative form of the parasite in mammalian cells. After several rounds of replication, the amastigotes differentiate into another infective form of the parasite, the bloodstream trypomastigotes, which are released upon rupture of the host cell membrane and infect neighboring cells or enter the bloodstream (Cardoso et al., 2015). Long-term persistence likely involves episodic reinvasion as well as continuous infection that extends to different tissues (Lewis and Kelly, 2016). Once the trypomastigotes reach the bloodstream, the parasite bypasses the complement system mediated lysis and opsonization, with the support of surface proteins (Cardoso et al., 2015) or by capturing host plasma molecules (Pangburn, 2000). Thus, the parasite disseminates through the bloodstream to many tissues during the acute phase that may veer toward a chronic phase. During this stage, about 30% of those infected will develop symptoms of this disease (Cardoso et al., 2015).

*Trypanosoma cruzi* infection activates both innate and adaptive immune responses during the acute phase. These responses involve complement system activation, macrophages, natural killer cells, B cells, CD4^+^ and CD8^+^ T cell activation, as well as the production of pro-inflammatory cytokines such as interferon-gamma, tumor necrosis factor, interleukin-12 and interleukin-17 (Cutrullis et al., 2017). Despite its activation, the immune response fails to completely eliminate the parasite, because *T. cruzi* has acquired a variety of strategies to evade the host immune response. Thus, trypomastigotes possess membrane-bound proteins that provide them with a certain degree of resistance to the complement-mediated lysis, enabling them to survive for longer periods in the bloodstream (Tomlinson and Raper, 1998). Early studies have demonstrated that trypomastigotes are highly complement resistant, while non-infective epimastigotes are highly susceptible (Muniz and Borriello, 1945; Rubio, 1956). This strategy to resist the complement system activation could be extremely important to define the evolutionary spectrum of possible hosts. Thus, birds are refractory to *T. cruzi* infection (Minter-Goedbloed and Croon, 1981; Teixeira et al., 2006). After inoculation, parasites cannot be recovered from bird blood (Teixeira et al., 1987). However, parasites inoculated to fertile chicken eggs infect embryo cells until the 10th day post-fertilization (Nitz et al., 2004). Thus, the elimination of the parasite could depend, in an important part, on the innate immune system and its maturation. Other studies indicate that metacyclic trypomastigotes are lysed by avian serum (Lima and Kierszenbaum, 1984), in an antibody-independent manner (Kierszenbaum et al., 1981). The administration of normal chicken serum to *T. cruzi* infected mice generates a marked decrease in their parasitemias (Kierszenbaum et al., 1976). These results indicate that, in addition to the maturation of the immune response, the avian serum and its complement system destroy the parasite. However, the molecules and mechanisms involved are unknown.

## Complement System Regulation in Pathogen Resistance to Complement

The complement system consists of about 40 proteins circulating in the blood/plasma or anchored to cell surfaces. This system is an important component of innate immune response which recognizes microbial pathogens, promoting opsonization, inflammation, clearance of immune complexes and cellular lysis (Kemper and Hourcade, 2008). The complement system recognizes different patterns on the aggressors or antibodies bound to pathogenic antigens. Three different complement system activation pathways have been identified: Classical (CP), activated by antigen:antibody complexes and other molecular patterns which are recognized by C1q sometimes in conjunction with adaptor molecules, such as C-reactive protein (Jiang et al., 1991); Lectins (LP), which responds to mannose containing polysaccharides through the collectin Mannan Binding Lectin (MBL), and to other targets such as acetyl sugars via the Ficolins and other Collectins such as CL-11 (Garred et al., 2016); AP, activated by a variety of microbial surfaces (Kemper et al., 2010). The activation of these three pathways concludes the formation of C3 and C5 convertase enzymes (Walport, 2001a,b), and generation of opsonins C3b and C4b (Kemper et al., 2010). In the CP, C1s, in the context of C1, a complex of three proteins C1q, C1r, C1s, is activated by binding of C1q to a target. C1s, a protease, will cleave both C4 and C2, forming the cell surface- bound C3 convertase (C4b,2b) that will activate C3, generating C3a and C3b fragments. C3b, similar to C4b, has a highly reactive thiolester bond that mediates binding to a nearby C4b,2b, thus initiating the assembly of C5-convertases (C4b,2b,3b), which cleaves C5 to generate C5a and C5b (Walport, 2001a,b; Kemper et al., 2010). In the C5-convertas both C4 and C3 are covalently bound to the target membrane. Then, C5b associates with C6 and C7 in the fluid-phase (plasma), resulting in the C5b-7 complex, which inserts into the membranes of target cells where it binds to C8 and C9, forming a MAC. This step from C5-C9 is also named the TP (Kemper et al., 2014). This MAC complex promotes membrane disruption and cell lysis (Walport, 2001a,b; Kemper et al., 2010). In the LP, the danger recognition lectin modules, Ficolins and MBL, will recognize a variety of pathogen associated molecular patterns (PAMPS or danger signals), with subsequent activation of their serine proteases (MASPs). The following activation steps are similar to those of the CP. The AP is activated by default, detecting the lack of certain molecules (e.g., sialic acid) that are prominent on the host cell surface. C3 (C3bBb) and C5 (C3bBbC3b)-convertases are generated. Given the structural similarities that exist between C3 and C4, and between C2 and factor B, the AP and CP convertases share extensive properties such as the capacity to cleave C3 and C5. The steps and molecules involved in the TP are common to all activation routes.

The complement system is tightly controlled by regulatory proteins present in plasma and on the cell membranes (Morgan, 2010), including those of selected pathogens. Thus, the CP is regulated by C1-INH, a serine protease inhibitor in plasma that binds activated C1, removing C1r and C1s from the complex and inactivating them (Davis, 1988, 1989). The AP downregulation is provided by FI, a serine protease that cleaves C3b in the convertases (Morgan, 2010). In fluid-phase, there are two proteins acting as cofactors for FI: FH and C4BP (Vik et al., 1990). Both break up convertases, a property termed “*decay acceleration”* (Gigli et al., 1979; Reid and Turner, 1994). On membranes, decay-accelerating factor protein (DAF) binds to and disassemble the CP and AP convertases. The membrane cofactor protein (MCP) acts as a cofactor for the cleavage of C4b and C3b by FI. Another cell-surface protein, complement receptor 1 (CR1) inactivates CP and AP convertases, causing dissociation and acting as a FI cofactor. On the other hand, the AP possesses a protein that stabilizes the C3 and C5 convertases, named Properdin (P), the only positive complement regulatory protein described so far (Smith et al., 1984). This protein is also a danger recognition module for pathogens such as *Chlamydia pneumonia* (Cortes et al., 2011) and *Neisseria gonorrhoeae* (Spitzer et al., 2007).

Other complement regulatory proteins, present in the fluid-phase and on membranes, tightly regulate MAC formation. The fluid-phase C5b-7 complex is a target for S-protein and clusterin, abundant serum proteins. On the membrane, CD59 binds to C8 in the C5b-8 complex and blocks the incorporation of C9, preventing MAC formation (Lachmann, 1991; Davies and Lachmann, 1993; Morgan, 2010).

All three complement pathways can be activated in the absence of antibodies, and so the complement system acts as the first barrier preventing infection. However, certain microorganisms have developed mechanisms to escape attack and survive in the host bloodstream (Iida et al., 1989). Thus, particular pathogens possess complement regulatory proteins on their surfaces or recruit them from host plasma, as a strategy to resist the complement activation. In *T. cruzi*, several complement regulatory proteins have been described. Some of them have been known for many years (Tomlinson and Raper, 1998) while others have recently been revealed.

## Different Stages and Strains of *T. cruzi* Present Variable Susceptibility to the Complement System

During its life-cycle, *T. cruzi* experiences multiple regulated morphological and physiological modifications to survive within the triatomine vector and mammalian host cells. Thus, bloodstream trypomastigotes (infective form in mammalian host), amastigotes (intracellular replicative stage in host cells) and metacyclic trypomastigotes (infective forms present in the vector’s feces) resist the complement-mediated lysis and the macrophage defense mechanisms. Differently from trypomastigotes, epimastigotes, the replicative and non-infective form of the parasite, present in the mid-gut of triatomine vectors, are highly susceptible to the complement-mediated lysis and cellular destruction (Muniz and Borriello, 1945; Rubio, 1956; Cestari Idos et al., 2008).

Early experiments indicated that mice infected with virulent bloodstream trypomastigotes present a marked exacerbation of *T. cruzi* infection with higher parasitemia and mortality when the complement system is depleted with cobra venom factor (Budzko et al., 1975). This finding indicates that complement is important to control the acute infection. The complement system may cause lysis by activation of the cascade or facilitate the parasite destruction by phagocytic cells. Infective forms of *T. cruzi* (trypomastigotes) in the presence of specific antibodies plus complement, show a certain proportion of surviving parasites (Kierszenbaum and Ramirez, 1990). In an attempt to select a trypomastigote subpopulation with increased resistance to lysis by specific antibodies plus complement, the parasites sensitive to immune lysis were eliminated by three successive passages in mice. The resulting parasites maintained their level of sensitivity and produced similar levels of parasitemia (Kierszenbaum and Ramirez, 1990). This finding indicates that the complement resistance is more dependent on phenotypic than genetic variations. In other words, it is likely that the number of complement regulatory proteins on the parasite surface follows a phenotypic normal distribution, and this will affect their complement system susceptibility. Consequently, the ability to resist the complement differs not only among different *T. cruzi* strains (Cestari Idos et al., 2008), but also among individuals in the same strain. Thus, the Colombiana (TcI) strain is more susceptible than the Y strain (TcII). Lysis is apparently due to the CP and LP in early stages of activation (Cestari Idos et al., 2008).

The role of complement in *T. cruzi* immune lysis has been studied for a long time. This susceptibility varies considerably, among other factors, with the parasite developmental stage (Anziano et al., 1972). During its cycle in the insect, *T. cruzi* assumes various forms and its infectivity is associated with the acquisition of resistance factors (Iida et al., 1989). As previously mentioned, while metacyclic and bloodstream trypomastigotes are resistant, the epimastigotes are extremely sensitive to the complement system (Muniz and Borriello, 1945; Rubio, 1956). Amastigotes, the replicative form of the parasite, are not exclusively intracellular, being found in the bloodstream during the infection acute stages (Ley et al., 1988). In circulation, amastigotes are extremely efficient activators of the complement system cascade, binding the terminal components (C9 polymerization). Although this parasite stage activates the AP, lysis does not occur because the C5b-9 complexes bind but do not insert into the lipid bilayer of the plasma membrane (Iida et al., 1989). This kind of activation in the absence of final lysis also occurs in other parasites such as promastigotes of *Leishmania,* which activate the complement system, but with a minimal surface deposition of C9 (Puentes et al., 1988). Bacteria such as *Borrelia burgdorferi* produce an 80 kDa CD59-like protein, which has affinity for C8β subunit and C9, and prevent MAC formation (Pausa et al., 2003; Lambris et al., 2008). A possible explanation of amastigote resistance to the complement system is that hydrophobic domains present on parasite surface molecules serve as non-specific “traps” for nascent C5b-7 complexes that will inefficiently bind C8 and C9. Another possible explanation is the presence of an unidentified specific inhibitor, homologous to a host factor that will prevent the incorporation of C8 and C9 on amastigotes (Iida et al., 1989). This resistance, both in trypomastigotes and amastigotes, involves the expression of surface and soluble components playing a role in complement activation.

## Complement Regulatory Proteins Expressed on *T. cruzi* Trypomastigotes

**FIGURE 1:**
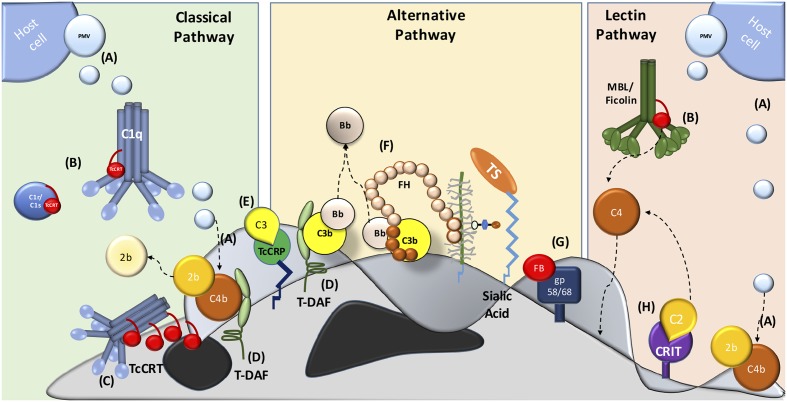
Major strategies involved in host complement system evasion by *T. cruzi*. The three complement pathways are inhibited by *T. cruzi* trypomastigotes. CP inhibition: **(A)** plasma membrane derived vesicles (PMV), inactivating the C3 convertase; **(B,C)** TcCRT, binding to C1q, C1r and C1s and inactivates the C1 complex; **(D)** T-DAF and **(E)** TcCRP, inactivating the C3 convertase. AP inactivation: **(D)** T-DAF, **(E)** TcCRP, **(F)** FH and **(G)** gp 58/68, inhibiting the C3 convertase formation. LP inhibition: **(A)** PMV, inactivating the C3 convertase; **(B)** TcCRT, binding to MBL and L-Ficolin and **(H)** CRIT, binding and inactivating C2.

Several molecules present on the parasite mediate resistance at different levels of the complement system cascade. In addition, trypomastigotes capture inhibitory host components, which are used to inhibit complement activation on the parasite surface, such as: PMV (**Figure 1A**) (Cestari et al., 2012), TcCRT (**Figures 1B,C**) (Ferreira et al., 2004), *T. cruzi* trypomastigote-decay accelerating factor (T-DAF) (**Figure 1D**) (Kipnis et al., 1988; Tambourgi et al., 1993), *T. cruzi* complement regulatory protein (TcCRP) (**Figure 1E**) (Norris et al., 1991; Norris and Schrimpf, 1994; Beucher et al., 2003); FH (**Figure 1F**) (Schenkman et al., 1986), gp58/68 (**Figure 1G**) (Fischer et al., 1988) and C2 receptor inhibitor trispanning (CRIT) (**Figure 1H**) (Cestari Idos et al., 2008). The molecular inhibitory mechanisms of these proteins are only partially known for some of these molecules, but the central role is in the inhibition (decay) of C3 and/or C5 convertases. The inhibition of these key enzymes may have important biological consequences, such as: (i) inhibition of complement-mediated lysis, (ii) decrease in C3a and C5a (anaphylatoxins) generation (these small complement fragments are essential in the recruitment of blood cells to the infection site), and (iii) decreased opsonization, which mediates phagocytosis of pathogens during the infection (Cestari et al., 2012).

### Trypomastigote Decay-Accelerating Factor (T-DAF)

Human DAF, a 70 kDa glycophospholipid-anchored membrane protein that regulates the CP and AP C3 and C5 convertases, is present on human erythrocytes, neutrophils, lymphocytes, monocytes, platelets and ECs, protecting them from autologous complement (Fujita et al., 1987).

*Trypanosoma cruzi* trypomastigotes (metacyclic, bloodstream and tissue culture-derived), but not epimastigotes, express a surface glycoprotein of 87–93 kDa, with decay accelerating activity, named trypomastigote-decay accelerating factor (T-DAF) (Kipnis et al., 1988; Tambourgi et al., 1993). T-DAF interferes with the efficient assembly of the C3 and C5 convertases of both CP and AP (**Figure 1D**), and it is functionally analogous to human DAF (Tambourgi et al., 1993). Patients chronically infected with *T. cruzi* have antibodies against this protein (Joiner et al., 1988; Tambourgi et al., 1989). The isolation of a partial cDNA clone of T-DAF and its deduced protein sequence showed 40% homology with a portion of the coding region for human DAF (Tambourgi et al., 1993). Human DAF activity is species restricted, while T-DAF protects the parasite against complement system from different mammal species (Tambourgi et al., 1993).

### *Trypanosoma cruzi* Complement Regulatory Protein (TcCRP)

*Trypanosoma cruzi* Complement Regulatory Protein (TcCRP) also named gp160, is a surface-anchored glycoprotein which inhibits the CP and AP (**Figure 1E**). TcCRP is expressed by trypomastigotes, but not by epimastigotes (Norris et al., 1991). However, stable TcCRP-transfected epimastigotes are protected from complement-mediated lysis, suggesting that epimastigote TcCRP expression is sufficient to confer a virulence-associated trait (Norris, 1998). First, TcCRP was purified as a 160 kDa glycoprotein present on trypomastigotes with complement regulatory activity. TcCRP binds C3b and C4b (Norris et al., 1991; Norris and Schrimpf, 1994; Norris, 1998) and inhibits the CP and AP C3 convertase formation. This protein is anchored to the trypomastigote membrane via a glycosyl phosphatidylinositol linkage similar to human DAF, but with only two possible SCR and significant DNA sequence identity with human DAF. This protein is a member of the C4/C5 binding family of complement regulatory proteins. Apparently, TcCRP has a decay accelerating activity (inhibiting the C3 convertase), but is not a cofactor for FI (cofactor activity) (Norris et al., 1991).

The *T. cruzi* genome contains multiple copies of TcCRPs, and proteins encoded by these genes share sequence similarity with members of the *T. cruzi*-TS superfamily. Since *T. cruzi* requires sialic acid to survive in the mammalian milieu, TS, parasite proteins with enzymatic capacity, transfer monosaccharide from host sialyl-glycoconjugates to terminal β-galactoses of acceptor molecules located on its own surface. These proteins display a variety of biological functions, many of them independent of their enzymatic activities, which promote the evasion of immune response (Previato et al., 1985; Freire-de-Lima et al., 2015). The TS- superfamily is classified by genomic analysis in eight groups, where only group-I has enzymatic activity, and groups II-VIII are considered inactive (Freitas et al., 2011). Within the TS superfamily, TcCRP belongs to a subfamily that lack TS activity (Beucher and Norris, 2008). Recently, a positive correlation between the virulence of *T. cruzi* strains and TcCRP expression levels has been described (Henrique et al., 2016).

### *Trypanosoma cruzi* Complement C2 Receptor Inhibitor Trispanning Protein (CRIT)

First discovered in *Schistosoma* and humans, CRIT is a 32 kDa protein, containing a 27 amino acid extracellular domain, highly similar to the C4β-chain (Inal and Sim, 2000; Inal and Schifferli, 2002; Inal, 2005). This protein is a C2 receptor present on *T. cruzi* and inhibits C2 cleavage by C1s (**Figure 1H**) (Inal and Schifferli, 2002).

C2 Receptor Inhibitor Trispanning Protein (CRIT) from the trypomastigote Y strain binds to and inactivates C2 (Cestari Idos et al., 2008). *T. cruzi* activates the LP, where a rapid binding of MBL, H-Ficolin, and L-Ficolin to the surface of *T. cruzi* is detected (Cestari Idos et al., 2008). In addition, complement sensitive epimastigotes, activate the LP, with MBL, L-Ficolins and H-Ficolins binding. These components plus C2 and C4 are required for the epimastigote-mediated lysis (Cestari Idos et al., 2009). MBL, Ficolins and the MASP-2 enzyme bind to metacyclic trypomastigotes in early stages of complement activation (Cestari Idos et al., 2009). How MBL binds to and interacts with trypomastigotes is still unknown (Cestari Idos et al., 2009). However, trypomastigotes that express CRIT inhibit LP activation and complement-mediated killing (Cestari Idos et al., 2008). Transgenic parasites overexpressing CRIT are highly resistant to complement-mediated lysis (Cestari Idos et al., 2009), where a specific CRIT extracellular domain 1 inhibits the C2 cleavage by MASP2 and impairs C3 convertase formation (Cestari Idos et al., 2009).

CRIT is expressed in different parasite strains (Cestari Idos et al., 2008). The gene sequences from other strains such as Cl Brenner, Colombiana and Dm28c are highly similar (88, 90, and 98% identity, respectively) (Cestari Idos et al., 2008), indicating related function in these strains. However, apparently the CRIT gene is only functional in some *T. cruzi* linages. Thus, when a TcII strain is transfected with the CRIT gene from a TcI strain, the susceptible strain TcII becomes more resistant to the complement-mediated lysis (Cestari Idos et al., 2008).

### Glycoprotein 58/68

Gp 58/68 is a 58 kDa glycoprotein (as assessed by SDS-PAGE in unreduced condition and 68 kDa, in a reduced condition) present on trypomastigotes (Velge et al., 1988). Gp58/68 inhibits the AP C3 convertase (decay-accelerating activity) (**Figure 1G**). However, unlike other complement regulatory proteins, such as T-DAF and FH, gp58/68 does not have cofactor activity (Fischer et al., 1988).

## A Fluid-Phase Host Complement Regulatory Proteins, FH, Is Used by *T. cruzi* to Evade Complement Activation

Trypomastigotes cover their surface with sialic acid residues taken from the host by TS (Frasch, 2000). Sialylated molecules downregulate AP activation (Pangburn et al., 2008; Zipfel and Skerka, 2009). FH, the AP negative regulatory protein, could also play a role on parasite complement resistance. FH is composed of 20 SCR domains (Pangburn, 2000) with different functions. FH possesses three sites (Sharma and Pangburn, 1996) that interact with unique domains on C3b (Alsenz et al., 1985; Lambris et al., 1988; Sharma and Pangburn, 1996; Jokiranta et al., 2000), participating in: (i) decay-accelerating activity for the AP C3/C5 convertases, (ii) cofactor for FI activity, and (iii) interaction with sialic acids (Alsenz et al., 1984; Kuhn et al., 1995; Pangburn, 2000). *T. cruzi* possesses TS to transfer α(2-3)-linked sialic acid from serum glycoconjugates to acceptor sites on the parasite surface (Schenkman et al., 1991; Tomlinson et al., 1994). These glycoconjugates restrict complement activation on the microbes surface, and are considered a virulence factor (Edwards et al., 1982; Marques et al., 1992; Wetzler et al., 1992; Edwards et al., 1993). This polyanion on the trypomastigote surface may also be critical for parasite survival in the circulation (Kipnis et al., 1981; Sher et al., 1986). In parallel, FH binds to *T. cruzi*, with higher affinity to metacyclic trypomastigotes than to epimastigotes (Schenkman et al., 1986). Since, FH binds to trypomastigotes covered by sialic acid via TS action, it could participate as an important complement regulatory protein to control the AP activation (**Figure 1F**).

## Plasma Membrane-Derived Vesicles (PMVs) and *T. cruzi* Exosomes

Plasma membrane-derived vesicles play important roles in several infectious- and non-infectious diseases and stress (Combes et al., 1999, 2005; Al-Nedawi et al., 2008; Bebawy et al., 2009; Faille et al., 2009; Ansa-Addo et al., 2010). Bloodstream and ECs release PMV from their cellular membranes (Gasser et al., 2003; Pilzer et al., 2005; Coltel et al., 2006; Ansa-Addo et al., 2010). *T. cruzi* trypomastigotes are exposed to PMVs from several cell types. *T. cruzi* induces an increase in PMV released from blood cells in a Ca^2+^-dependent manner. *T. cruzi* surface molecules such as gp82 and oligopeptidase B induce a transient increase of intracellular Ca^2+^ in host cells, which could induce PMV release. These vesicles bind to C3 convertase, inhibiting its catalytic activity of both CP and LP (**Figure 1A**). The molecules on the PMVs interacting with C3 convertases are still unknown (Cestari et al., 2012). On the other hand, TGF-β-bearing PMVs promote host cell invasion via the lysosome-independent route. This phenomenon is dose- and parasite- infective stage dependent and non-specific for parasite strains or host cell types (Cestari et al., 2012). In agreement with this TGF-β function, chronically infected patients have higher levels of circulating TGF-β (Araújo-Jorge et al., 2002). Thus, TGF-β-bearing PMVs could activate the TGF-β signaling pathway to promote parasite infectivity (Cestari et al., 2012). Similar PMVs-mediated mechanisms have been described to other infectious agents such as HIV (Mack et al., 2000) and *Plasmodium* sp. (Faille et al., 2009). At the same time, *T. cruzi* produces exosomes that stimulate different host cells to produce PVMs to modulate the host immune response (Cestari et al., 2012; Mantel and Marti, 2014). The *T. cruzi* exosomes contain several surface components, like glycoproteins, such as gp85/transialidases, alphaGal-containing molecules, proteases, cytoskeleton proteins, mucins, and associated to GPI-anchored molecules.

## *Trypanosoma cruzi* Calreticulin, an Important Virulence Factor

Our laboratory has been working for over 20 years with TcCRT, a multifunctional ER-resident protein that the parasite translocates to the external milieu, mainly via the area of flagellum emergence. Although TcCRT is located mainly in the ER, histochemical studies have found it also in the Golgi, reservosomes, flagellar pocket, cell surface, cytosol, nucleus and kinetoplast (Ferreira et al., 2004; Souto-Padron et al., 2004; Gonzalez et al., 2015). However, the mechanisms involved in these heterogeneous TcCRT locations are still unknown.

Similar to its human counterpart HuCRT, TcCRT is a key protein for *T. cruzi*. Thus, epimastigotes bearing a monoallelic deletion of the TcCRT gene present a reduced rate of differentiation to trypomastigotes in an *in vitro* assay (Sanchez-Valdez et al., 2013).

**FIGURE 2:**
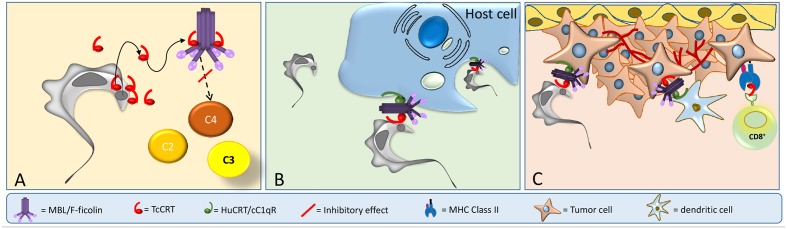
TcCRT participates in three important mechanisms related to *T. cruzi* infection. **(A)** TcCRT, translocated and secreted by the parasite, binds to C1 or MBL or L-Ficolin inactivating the CP and LP of complement system. **(B)** TcCRT binds C1 on the area of flagellar emergence. The TcCRT/C1q complex is recognized by CRT on the host cell surface (cC1qR), promoting host cell infectivity. **(C)** TcCRT has anti-angiogenic and anti-tumor growth properties.

In spite of the long evolutionary distance, TcCRT shares 50% homology with HuCRT (Ramirez et al., 2011a), a molecule with more than 40 functions described (Eggleton et al., 2016). HuCRT has three domains: globular N-terminus (N), proline-rich (P), and acidic C-terminus (Michalak et al., 2009). An S-domain (aa 160–283), within N and P, interacts with C1q (Stuart et al., 1996, 1997), the first component of the CP. TcCRT shares these domains and is involved in a variety of host-pathogen interactions, such as immune evasion, *T. cruzi* infectivity and tumor growth inhibition (**Figure 2**).

### TcCRT Participates in the Inhibition of *T. cruzi* Complement-Activation

*Trypanosoma cruzi* inhibits the early stages of CP activation. Previous reports indicate that the CP is activated inefficiently on *T. cruzi* and it is rendered more efficient in the presence of specific lytic antibodies present in *T. cruzi* seropositive individuals (Almeida et al., 1994a,b,c). Other studies indicate that the CP participates as an enhancer for the AP in *T. cruzi* complement activation because C2 depletion or CP inhibition (using EGTA) do not affect significantly the parasite complement-mediated lysis (Krettli et al., 1979). The LP has a key role in host defense. Mutations in LP main components, MBL, Ficolins and MASPs, increase susceptibility to several infectious diseases (Hummelshoj et al., 2005; Eisen et al., 2008). The LP is activated on *T. cruzi*. This parasite binds MBL, *H*-Ficolin and L-Ficolin and serum depleted of these molecules failed to destroy the parasites (Cestari Idos et al., 2009). LP activation also triggers AP activation by C3b generation. This synergic effect enhances the complement-lysis capacity (Cestari Idos et al., 2009). One report, however, indicates that MASP-2 full deficiency in KO mice, does not increase mice susceptibility to *T. cruzi* (Ribeiro et al., 2015).

In this context, our laboratory has shown that TcCRT is involved in several key aspects of the host/parasite interplay, such as inhibition of both the CP and LP. TcCRT inhibits the CP, interacting with C1 (**Figures 1B**, **2A**). This interaction inactivates the CP at the earliest stages. TcCRT binds to the collagenous tails of C1q interfering with the activation of C1s and subsequent C1s-mediated cleavage of C4 and, as a consequence, inhibiting the entire cascade (Ferreira et al., 2004). The capacity to bind C1q and inhibit complement resides in the central TcCRT S-domain (aa 159–281). TcCRT competes with the (C1r-C1s)_2_ tetrameric complex to bind C1q, decreasing C4b generation and, as a consequence, the levels of the CP C3 and C5 convertases generated (Ferreira et al., 2004). The TcCRT capacity to bind C1q and to inhibit C4b generation is calcium-independent (Valck et al., 2010). In addition to C1q, both CP serine-proteases, C1s and C1r, bind TcCRT *in vitro*. However, TcCRT does not inhibit the C4-activating function of solid phase-bound C1s. Perhaps, C1s inactivation occurs only when the serine protease is part of C1 complex [C1q, (C1r, C1s)_2_] (Valck et al., 2010). Additionally, TcCRT competes with the serine proteases, but does not displace them from the preformed C1 complex (Valck et al., 2010).

Several functions are shared and conserved to differing degrees by calreticulin (CRT) from different species (Ramirez et al., 2011a). Recently, an important domain of *Triatoma infestans* calreticulin (TiCRT), protein present in saliva of this vector, was cloned and expressed. This domain also binds C1, inhibiting the CP. The presence of TiCRT in hematophagous triatomines saliva may control the potential activation of complement in the digestive tract of these vectors and subsequent tissue damage (Weinberger et al., 2016). This function has been studied in other species, such as *Amblyomma americanum*. This tick, while feeding on its host, secretes CRT (Jaworski et al., 1996), presumably as a mechanism to evade or inactivate the host complement system (Ramirez et al., 2011a), thus preventing damage to its digestive mucosa.

A recent crystallographic structural study defines CRT conformational rearrangements that could be informative in future therapeutic investigations of parasite CRTs (Moreau et al., 2016), mainly in its anti-complement and anti-neoplastic effects.

TcCRT also binds MBL and Ficolins, inhibiting the LP (**Figure 1B**) (Sosoniuk et al., 2014). *L*-, *H*- and *M*-Ficolins have been described (Endo et al., 1996; Lu et al., 1996; Sugimoto et al., 1998; Teh et al., 2000; Wittenborn et al., 2009). *L*- and *H*-Ficolins bind to LTA (Lynch et al., 2004) and acetylated sialic acids, both cell components found in Gram-positive and -negative bacteria, respectively, with subsequent MASP activation (Gout et al., 2010). However, L-Ficolin (but not *H*-Ficolin) binds to TcCRT, but the TcCRT/L-Ficolin does not interfere with LTA binding to L-Ficolin, but does interfere with its activation via LTA. Moreover, L-Ficolin binds preferentially to trypomastigotes, as compared to epimastigotes, which translocate significantly lower amounts of TcCRT to their surfaces (Sosoniuk et al., 2014). Whether *M*-Ficolin binds is still unknown.

Furthermore, parasites carrying a monoallelic deletion of the *TcCRT* gene, are significantly susceptible to complement-mediated lysis. As expected, parasites overexpressing TcCRT are significantly more resistant to the complement-mediated lysis by CP and LP (Sanchez-Valdez et al., 2013, 2014).

### TcCRT and Its Role in the *T. cruzi* Infectivity Process

CRT is also a membrane receptor for C1q, named cC1qR (Eggleton et al., 2000). This protein participates in the immune response against apoptotic cancer cells, as an *“eat me”* signal required for phagocytosis on dying tumor cells (Gardai et al., 2005). Apoptotic cells expose CRT on their surfaces, where it is recognized by C1q. The CRT/C1q complex on the parasite is an *“eat me”* signal for phagocytic cells expressing cC1qR (Malhotra, 1993).

TcCRT, expressed mainly in the area of parasite flagellum emergence, recruits C1q/C1, in a molecular mimicry strategy. Similar to apoptotic cells, the TcCRT/C1q complex is recognized as an *“eat me”* signal by cC1qR/CRT on phagocytes and other cellular types, thus promoting infectivity (**Figure 2B**). As expected, anti-TcCRT F(ab’)_2_ antibodies inhibit the TcCRT/C1q interaction and, as a consequence, decrease infectivity *in vitro* and *in vivo* (Ramirez et al., 2011b). Additionally, this TcCRT/C1q-mediated infectivity correlates with a significant increase in TcCRT mRNA levels in the early infection steps (Ramirez et al., 2011b). Unlike the infective forms, epimastigotes are highly sensitive to the complement activation, most likely due, at least in part, to the marginal levels of TcCRT expressed on their surfaces (Ferreira et al., 2004; Abello-Caceres et al., 2016). However, epimastigotes bind exogenously added TcCRT and are internalized by fibroblasts in a C1q-dependent manner. Likewise, CRT-deficient fibroblasts have impaired capacity to internalize TcCRT, indicating again that the TcCRT/C1/CRT complex participates in the invasion process (Sosoniuk-Roche et al., 2016). Mice inoculated with mutant trypomastigotes (carrying a monoallelic TcCRT deletion gene), do not generate detectable parasitemia, meaning that parasites do not proliferate and therefore no anti-*T. cruzi* IgG antibodies are generated. Thus, these mutants are restricted in two important properties conferred by the TcCRT/C1q interaction: their capacity to evade the CP and LP (**Figure 1B**), and their virulence (**Figure 1C**) (Sanchez-Valdez et al., 2014).

This infection strategy is important in the congenital transmission of trypanosoma infection, given its relevance in the epidemiology of this infection, in particular its emergence in developed countries. The human placenta expresses high CRT levels (Houen and Koch, 1994), in particular during pregnancy (Gu et al., 2008; Crawford et al., 2012). Translocated parasite TcCRT, binds and inactivates C1 that will in turn bind the parasite to cC1qR present on the placental ST. This newly formed TcCRT/C1q/CRT synapsis is important to infect the human placenta, as measured in an *ex vivo* model (Castillo et al., 2013). Thus, a molecular basis that explains at least an important part of the parasite to contact the ST is provided.

TcCRT also binds to MBL and L-Ficolin (Ferreira et al., 2004; Valck et al., 2010), but the role of TcCRT/MBL or L-Ficolin in *T. cruzi* infectivity process has not yet been addressed. However, a study using two *T. cruzi* strains, susceptible and resistant, suggested that MBL participates in the infectivity process while the parasite deactivates the LP (Evans-Osses et al., 2014), but the ligand for MBL on the parasite surface is still unknown. Besides the TcCRT interplays with MBL, C1q and L-Ficolin, additional studies on the possibilities of functional interactions with SP-A, SP-D should be undertaken.

### TcCRT Interactions with the Complement System Mediate Tumor Growth Inhibition

For several decades now an antagonism between Chagas’ disease and tumor development has been observed. Several *T. cruzi* strains exhibited growth inhibitory properties over transplanted or spontaneous tumors, in murine experimental models and in humans (Kallinikova et al., 2001; Oliveira et al., 2001). The molecular mechanisms or particular molecules involved in this process were unknown, although the anti-tumor property was attributed to a “toxic substance,” secreted by the parasite, which reduced pain, inflammation, tumor growth and bleeding (Roskin, 1946; Hauschka and Goodwin, 1948; Klyuyeva and Neish, 1963; Krementsov, 2002).

Our laboratory has contributed to elucidate the role of TcCRT as a mediator of at least an important part of this effect. TcCRT is anti-angiogenic *in vitro* (Lopez et al., 2010), *ex vivo* (Toledo et al., 2010) and *in vivo* (Lopez et al., 2010; Aguilar-Guzman et al., 2014; Abello-Caceres et al., 2016), inhibiting the growth of mammary adenocarcinoma and melanoma in murine models (Molina et al., 2005; Lopez et al., 2010; Toledo et al., 2010; Aguilar-Guzman et al., 2014). A recombinant TcCRT and its specific N-terminal domain (aa 120-180) inhibit capillary growth *ex vivo* in *Rattus rattus* aortic rings, morphogenesis, proliferation and chemotaxis in human umbilical cord ECs (HUVEC) (Lopez et al., 2010) and an *in vivo* angiogenesis model in *Gallus gallus* chorioallantoic membrane assay (Molina et al., 2005). Specific anti-TcCRT antibodies inhibit the TcCRT anti-angiogenic effect (Toledo et al., 2010). This effect is shared with HuCRT. However, TcCRT is approximately 2-fold more efficient at equimolar concentrations (Lopez et al., 2010; Toledo et al., 2010). In addition, TcCRT is internalized by ECs. However, this internalization is susceptible to be inhibited by Fucoidan, a ligand for SR (Lopez et al., 2010), indicating SR as an additional possible receptor to TcCRT on ECs. In addition, the interaction of TcCRT and ECs can be inhibited by anti-TcCRT antibodies or Fucoidan, thus strengthening the participation of SR in this interaction (Abello-Caceres et al., 2016). These antibodies also revert the TcCRT effect on EC proliferation (Abello-Caceres et al., 2016).

TcCRT also inhibits tumor growth. TcCRT inoculated in a peritumoral area reduced, more efficiently than HuCRT, the growth of an aggressive, multi-resistant mammary adenocarcinoma (TA3-MTXR) in an *in vivo* murine model (Lopez et al., 2010). Indeed, in these experiments HuCRT did not show antitumor effects. Since all these effects were tested using exogenous recombinant TcCRT, we recently verified the effect of native TcCRT (in the context of the parasite) on the tumor growth in infected animal. Thus, mice were inoculated with TA3-MTXR tumor cells and infected with *T. cruzi* trypomastigotes. In parallel, these animals were treated with anti-TcCRT antibodies. As expected, these antibodies, but not their pre-immune counterparts, neutralized the anti-tumor effect of the infection, indicating that native endogenous TcCRT, in the context of the parasite, is responsible for at least an important part of the anti-tumor effect of *T. cruzi* infection (Abello-Caceres et al., 2016). Thus, our model proposes that, native TcCRT (secreted or on the parasite) mediates fundamental alterations in the tumor cell microenvironment leading to an adaptive immune response, with significant anti-tumor consequences.

Once in the bloodstream, trypomastigotes infect ECs by recruiting C1 via externalized TcCRT (**Figures 2A,B**). This TcCRT also inhibits angiogenesis (**Figure 2C**) and allows the contact with EC via HuCRT [also known as cC1qR (Stuart et al., 1997)] or SRs (**Figure 2C**). Tumor cell phagocytosis by dendritic cells is stimulated through C1q recruitment (*“eat me”* signal). The evoked adaptive immune response could be stimulated by TcCRT (or HuCRT). Thus, dendritic cells will cross-present antigenic peptides derived from TcCRT (and/or from still unidentified tumor specific antigens-TSAs) to cytotoxic T cells in the draining regional lymph nodes (**Figure 2C**). These activated T lymphocytes will return to the tumor site and act against tumor cells (Ramirez-Toloza et al., 2016). The possible simultaneous role of T helper, NK and NKT cells are matters of current research in our laboratory. Mammal CRTs are about 95% identical among them, while TcCRT differs by 50% in its primary sequence, from these counterparts. Therefore, rTcCRT upon binding to tumor cells, is potentially more suitable than HuCRT to force their immunogenicity (Weinberger et al., 2016).

## Conclusion

*Trypanosoma cruzi* has developed different adaptive strategies to avoid destruction by the host immune system, in particular by complement. In many conditions, the parasite has acquired these abilities only at the stage of development that are infective for the host (trypomastigotes and amastigotes). Thus, the non-infective forms of the parasite are susceptible to the complement in the presence of human serum while trypomastigotes are highly resistant. Recently, it has been demonstrated that this resistance varies also between strains. The complement system is a physiological barrier during infection which the infective forms must evade in early stages. These infective forms express on their surfaces different complement regulatory proteins or capture them from the host plasma to inhibit the complement system. Most of these proteins have a decay-accelerating activity, inhibiting the C3 convertase formation (such as T-DAF, CRP, gp58/68 and FH) and/or participate as a cofactor for FI (such as FH). Other components inactivate the catalytic activity of C3 convertase (PMV), or bind and inactivate specific complement components (TcCRT and CRIT). Some of these proteins are multifunctional. Thus, TcCRT disrupts the initial step of CP and LP, reducing the C3 convertase formation. However, TcCRT is also an important virulence factor in association with C1. Thus, the TcCRT/C1 interaction on the parasite surface promotes infectivity *in vitro* and *in vivo.* On the other hand, native endogenous parasite TcCRT inhibits tumor development in infected animals, since this effect is fully reproduced by recombinant TcCRT and reversed by specific antibodies. This role is in agreement with previous reports indicating a possible protective role of *T. cruzi* infection in cancer development.

## Author Contributions

GR-T and AF designed and performed experiments, interpreted data, generated key reagents, wrote, revised, edited and approved the manuscript. GR-T designed the figures.

## Conflict of Interest Statement

The authors declare that the research was conducted in the absence of any commercial or financial relationships that could be construed as a potential conflict of interest.

## Abbreviations

AP: alternative pathway
C1-INH: C1-inhibitor
C4BP: C4 binding protein
CP: classical pathway
CRIT: *T. cruzi* C2 receptor inhibitor trispanning
CRT: calreticulin
DAF: human decay-accelerating factor
ECs: endothelial cells
ER: endoplasmic reticulum
FH: factor H
FI: factor I
gp58/68: glycoprotein 58/68
HuCRT: human calreticulin
LP: lectin pathway
LTA: lipoteichoic acid
MAC: membrane attack complex
MASPs: Mannan-binding lectin serine protease
MBL: Mannan-binding lectin
PAMPs: pathogen associated molecular patterns
PMVs: plasma-membrane derived-vesicles
SCR: short consensus repeats
SRs: scavenger receptors
ST: syncytiotrophoblast
TcCRP: *Trypanosoma cruzi* complement regulatory protein
TcCRT: Trypanosoma cruzi calreticulin
*T-DAF*: *Trypanosoma cruzi* trypomastigote decay-accelerating factor
TP: terminal pathway
TS: *Trans*-sialidase.
